# Promoter Methylation Status of Breast Cancer Susceptibility Gene 1 and 17 Beta Hydroxysteroid Dehydrogenase Type 1 Gene in Sporadic Breast Cancer Patients

**DOI:** 10.1155/2016/9545241

**Published:** 2016-06-20

**Authors:** Marwa M. Hosny, Nagwan A. Sabek, Taghrid B. El-Abaseri, Fathalla M. Hassan, Sherif H. Farrag

**Affiliations:** ^1^Department of Medical Biochemistry and Molecular Biology, Faculty of Medicine, Suez Canal University, Round Road, Ismailia 41111, Egypt; ^2^Surgery Department, Faculty of Medicine, Suez Canal University, Ismailia, Egypt

## Abstract

Epigenetic modifications are involved in breast carcinogenesis. Identifying genes that are epigenetically silenced via methylation could select target patients for diagnostic as well as therapeutic potential. We assessed promoter methylation of breast cancer susceptibility gene 1 (*BRCA1*) and 17 Beta Hydroxysteroid Dehydrogenase Type 1 (*17βHSD-1*) in normal and cancer breast tissues of forty sporadic breast cancer (BC) cases using restriction enzyme based methylation-specific PCR (REMS-PCR). In cancerous tissues,* BRCA1* and* 17βHSD-1* were methylated in 42.5% and 97.5%, respectively, while normal tissues had 35% and 95% methylation, respectively.* BRCA1* methylation in normal tissues was 12.2-fold more likely to associate with methylation in cancer tissues (*p* < 0.001). It correlated significantly with increased age at menopause, mitosis, the negative status of Her2, and the molecular subtype “luminal A” (*p* = 0.048, *p* = 0.042, *p* = 0.007, and *p* = 0.049, resp.). Methylation of* BRCA1* and* 17βHSD-1* related to luminal A subtype of breast cancer. Since a small proportion of normal breast epithelial cells had* BRCA1* methylation, our preliminary findings suggest that methylation of* BRCA1* may be involved in breast tumors initiation and progression; therefore, it could be used as a biomarker for the early detection of sporadic breast cancer. Methylation of* 17βHSD-1* in normal and cancer tissue could save patients the long term use of adjuvant antiestrogen therapies.

## 1. Introduction

Breast cancer is a malignancy arising from the epithelial tissues that line the terminal ductal-lobular units of the breast [1, 2]. Breast cancer (BC) is the most common cancer in females worldwide [3]; BC is the leading cause of cancer death among females, with an estimated 1.7 million cases and 521,900 deaths in 2012; BC alone accounts for 25% of all cancer cases and 15% of all cancer deaths among females [4]. Based on data from the National Cancer Registry Program of Egypt (NCRP) in 2008–2011, BC is the most common malignancy among Egyptian females. It constituted 32.0% of all cancer cases [5]. According to GLOBOCAN 2012, the age-standardized incidence rate (ASR) of breast cancer was 42.3 per 100,000 Egyptian females, with a mortality rate of 17.4 per 100,000 females [4].

Many environmental factors combined with multiple genetic and epigenetic changes are involved in the onset and development of breast cancer [6, 7]. The carcinogenesis process is a multistep process during which genetic and epigenetic alterations accumulate in a cell, resulting in the progressive transformation of normal cells through steps of initiation, promotion, and progression into cancer cells [8]. Epigenetics are emerging as one of the most important events in carcinogenesis [9]. DNA methylation has an essential role in the regulation of gene expression in mammalian cells [10]. In normal cells, the majority of promoter cytosine phosphate guanosine (CpG) islands are protected from this epigenetic event; thus, they are unmethylated. Conversely, in cancer cells, several promoter CpG islands are hypermethylated and form a closed repressive chromatin configuration that affects the transcription initiation of the corresponding genes [11–13]. Moreover, promoter methylation is a common epigenetic mechanism to silence genes during breast cancer development [14].

Currently, genes that are epigenetically regulated via promoter methylation in breast cancer include cyclin-dependent kinase inhibitor (*p16*), breast cancer susceptibility gene 1 (*BRCA1*), estrogen receptor (*ERα*), progesterone receptor (*PR*), retinoic acid receptor-*β*2 (*RARβ2*), glutathione S-transferase p1 (*GSTP1*), E-cadherin, and tissue inhibitor of metalloproteinase 3 (*TIMP3*) [15]. The identification of these methylated promoters had significantly contributed to elucidating the altered molecular pathways in breast carcinoma and provided potential targets for molecular detection [16].* BRCA1* is a tumor suppressor gene that is involved in critical biological processes, including DNA damage repair, cell cycle control, and transcriptional regulation [17]. Silencing of* BRCA1* gene via promoter hypermethylation is a common mechanism for its inactivation [18] ranging from 9 to 44% of sporadic breast cancers [19–21]. Breast cancer, with extensive hypermethylation in the* BRCA1* promoter, correlates with a reduced* BRCA1* expression [22]. In normal breast tissues,* BRCA1* promoter methylation had been identified in 8.3–22% [23].

The growth of both normal and neoplastic mammary tissue is affected by a number of hormones especially estrogen, which exists in several forms, estrone (E1), estradiol (E2), estriol, estrone sulfate, and estradiol sulfate. E2 is the most biologically active form in the breast tissue. Increasing evidence indicates that intratumoral estrogens derived in situ are mitogenic; thus they promote BC progression, irrespective of the serum concentrations of ovarian estrogen [24, 25].

17 Beta Hydroxysteroid Dehydrogenases (17*β*HSDs) catalyze the interconversion of active and inactive forms of estrogens within tissues. 17 Beta Hydroxysteroid Dehydrogenase Type 1 (17*β*HSD-1) mainly converts E1 to the potent E2. The encoding gene (17*β*HSD-1) is located at 17q12–21, a region that often is rearranged in breast cancer [26]. In a study carried out by Gunnarsson et al., they found amplification of the encoding gene (17*β*HSD-1) in 14.5% of the breast tumors [27]. Meanwhile 17 Beta Hydroxysteroid Dehydrogenase Type 2 (17*β*HSD-2) catalyzes the conversion of E2 to E1, thereby reducing estradiol level and hence controlling its proliferative activity [28]. The encoding gene (17*β*HSD-2) is located at 16q24 and loss of heterozygosity (LOH) at this site is frequent and early event in breast cancer [29]. Interestingly, BRCA1 had been found to negatively affect estradiol activity by direct interaction with the estrogen receptor, thus controlling the proliferation caused by this steroid hormone [30].

Clinical interest in the treatment of tumors has gained increased impetus interest because the evidence on the use of novel therapeutic agents suggests that DNA promoter methylation is potentially reversible. This may thus allow for the development of future therapeutic interventions [31]. In the light of these evidences and because of the potential roles of estrogens in the early stages of human breast carcinogenesis, in the present study, we aimed to assess the promoter methylation status of both* BRCA1* and* 17βHSD-1* genes in the tumor and adjacent normal tissue from sporadic breast cancer patients to establish the role of epigenetic in regulating intratissue estrogen activity and thereby in the etiology of breast cancer.

## 2. Material and Methods

### 2.1. Tissue Specimen Collection

Surgically resected specimens were freshly obtained at the operation room from forty diagnosed primary breast carcinomas enrolled in the Surgical Oncology Unit in Suez Canal University Hospital during the period from 2013 to 2014. Their matching normal breast tissues, taken 3–5 cm away from the healthy safety margin of the site of the tumor in the same breast, were obtained to serve as controls. Patients receiving neoadjuvant chemotherapy and/or hormonal therapy were excluded. Harvested breast tissues were either divided for DNA isolation or kept in 10% neutral-buffered formalin for histopathological analysis. Labeled tissue sections were examined by a pathologist blinded to the identity of samples and only the researcher would know to whom it referred. Prior to surgical tumor removal, written informed approval consents were obtained from all participants included in the study according to the Ethics Committee of the Faculty of Medicine, Suez Canal University.

### 2.2. Extraction of Genomic DNA

Genomic DNA was extracted from collected tissues using Qiagen Genomic DNA Purification Kit (cat # 51304; Qiagen, Hilden, Germany) according to the manufacturer's instructions. The quality of extracted genomic DNA was measured using the NanoDrop- (ND-) 1000 Spectrophotometer V3.1.0 (NanoDrop Technologies, Inc., Wilmington, DE, USA).

### 2.3. Restriction Enzyme Based Methylation-Specific Polymerase Chain Reaction (REMS-PCR)

**Figure 1:**
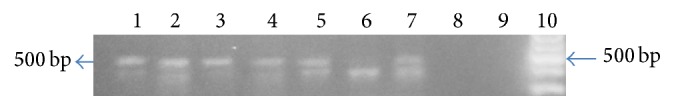
A representative gel picture of 2% agarose gel showing* BRCA1* methylation status of two cases. Lanes 1, 2, 3, 4, 5, and 7: the presence of (500 bp) band indicates methylation of* BRCA1* promoter in these specimens. Lanes 6 and 8: absence of (500 bp) band indicates the absence of methylation of* BRCA1* promoter in these specimens. Lane 9: negative control. Lane 10: 100 bp DNA ladder. Lanes 1 and 5: undigested DNA of normal breast tissue specimens; lanes 2 and 6: digested DNA of normal breast tissue specimens; lanes 3 and 7: undigested DNA of cancerous breast tissue specimens; and lanes 4 and 8: digested DNA of cancerous breast tissue specimens.

**Figure 2:**
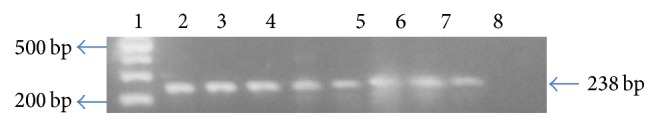
A representative gel picture of ethidium bromide-stained 2% agarose gel showing* 17βHSD-1* methylation status of two cases. Lane 1: a 100 bp DNA ladder. Lanes 2, 3, 4, 5, 6, 7, 8, and 9: the presence of (238 bp) band indicates methylation of* 17βHSD-1* promoter in these specimens. Lane 10: negative control. Lanes 2 and 6: undigested DNA of normal breast tissue specimens; lanes 3 and 7: digested DNA of normal breast tissue specimens; lanes 4 and 8: undigested DNA of cancerous breast tissue specimens; and lanes 5 and 9: digested DNA of cancerous breast tissue specimens.

Extracted DNA was digested using the fast restriction enzyme HpaII according to the manufacturer's instructions (Fast Digest®, Fermentas, CA, USA). The reaction mixture was incubated at 37°C in an oven for 1 hour followed by enzyme inactivation for 10 minutes at 90°C. Digested DNA aliquots were then PCR analyzed using* BRCA1* and* 17βHSD-1* specific primers [32] encompassing methylation-specific sites (Table 1) and the Taq PCR Master Mix Kit (cat # 201445; Qiagen, Hilden, Germany). A mock undigested sample containing 1 *μ*L of nuclease-free water, instead of 1 *μ*L of Fast Digest enzyme (HpaII), was used as a control. A sample of water instead of DNA was used as a negative control for each PCR. PCR was performed for 32 cycles using the following thermal cycling conditions: initial denaturation at 94°C for 5 minutes, denaturation at 94°C for 30 seconds, primer annealing at 55°C or 58°C for 30 seconds, extension at 72°C for 45 seconds, and a final extension at 72°C for 7 minutes. An attempt to perform* BRCAI* PCRs at higher annealing temperatures gave a total absence of bands. PCR products were detected using ethidium bromide-stained 2% agarose gels and bands were visualized under UV light. The presence of bands with sizes of 500 bp and 238 bp indicated methylation of* BRCA1* and* 17βHSD-1* promoters, respectively, while the absence of these bands indicated a lack of methylation (Figures 1 and 2).

### 2.4. Statistical Analysis

Coded collected data was analyzed using statistical package SPSS 16.0 for windows (SPSS, Chicago, IL, USA). Student's *t*-test was performed for statistical evaluation of quantitative variable between two independent groups in parametric data with *p* < 0.05 considered significant. Chi-square test, described in the form of frequency and percentages, was used to compare a qualitative variable between two independent groups.

## 3. Results

### 3.1. Association of* BRCA1* and* 17βHSD-1* Promoter Methylation with Clinicopathological Parameters

REMS-PCR showed PCR products of the expected sizes 500 bp and 238 bp for* BRCA1* and* 17βHSD-1* genes, respectively, in methylated samples, while the absence of these bands indicated a lack of methylation (Figures 1 and 2).

**Figure 3:**
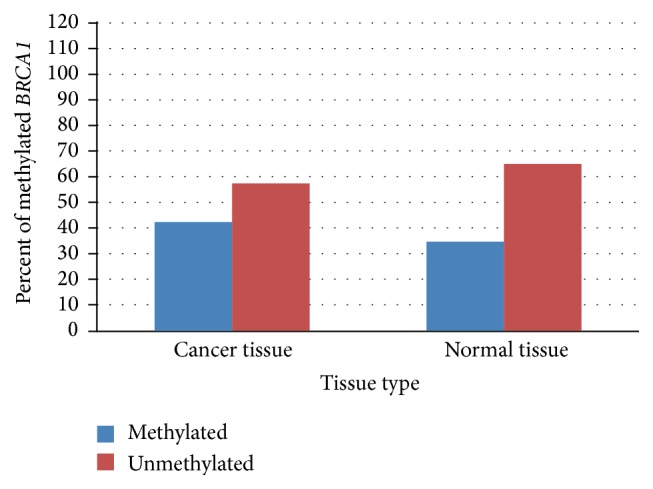
Difference between methylation status of* BRCA1* promoter in cancer and normal tissue specimens.

**Figure 4:**
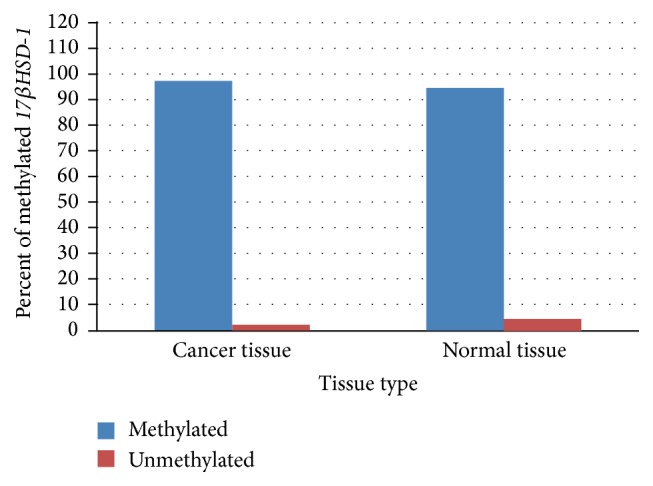
Difference between methylation status of* 17βHSD-1* promoter in cancer and normal tissue specimens.

*BRCA1* promoter was methylated in 42.5% of tumor tissue compared to 35% in control normal tissue (Figure 3). To our surprise,* 17βHSD-1* promoter methylation was seen in 97.5% of tumor tissues as compared to 95% of neighboring normal tissue (Figure 4). We found a trend towards* BRCA1* and* 17βHSD-1* promoter hypermethylation in cancer tissue specimens of sporadic breast cancer patients compared with controls, although the difference was nonsignificant (*p* > 0.05).

We categorized our patients into two age groups below 50 years and above or equal to 50 years; there was a nonsignificant increase in* BRCA1* promoter methylation of breast cancer tissue specimens in older women compared with younger patients (70.6% versus 29.4%, resp.) (Table 2). Similarly,* 17βHSD-1* promoter methylation, in both study groups, was not associated with age (Table 3), indicating no dramatic effect of age on* BRCA1* and* 17βHSD-1* methylation status. Moreover, postmenopausal status was associated with a nonsignificant increased methylation of* BRCA1* promoter in cancer tissue specimens compared with premenopausal females (70.6% versus 29.4%), respectively (Table 2). In contrast, the mean age at menopause was significantly higher among* BRCA1*-methylated than the* BRCA1*-unmethylated group in cancer tissue specimens, as shown in Table 2 (50.7 ± 3.1, 48.3 ± 3.4, resp.; *p* = 0.048). Finally, our results showed no statistically significant difference in* BRCA1* promoter methylation in both study groups with tumor size, the number of positive nodes, or tumor stage (Table 2). However,* BRCA1*-methylated promoter in cancer tissues tended to be of a higher grade (82.4% methylated versus 69.6% unmethylated in grade 2 “tumors”).

When studying* 17βHSD-1*, promoter methylation did not associate with any clinicopathological characteristics in both study groups (Table 3). In addition,* 17βHSD-1* promoter methylation did not correlate with lymph node status, clinical stage, or histological grade (Table 3).

### 3.2. Association of* BRCA1* and* 17βHSD-1* Promoter Methylation with Molecular Subtypes of Breast Cancer

Methylated* BRCA1* in breast cancer specimens correlated with increased mitotic index, the negativity of Her2 receptors, and hence molecular subtype “luminal A” (*p* = 0.042, *p* = 0.007, and *p* = 0.049, resp.) (Table 4). Although methylation of this promoter tended to be higher in positive estrogen receptor specimens, this correlation was not significant (Table 4).* 17βHSD-1* promoter methylation occurred with almost equal percentages when correlated with all molecular subtypes (Table 5). We observed a high trend towards* 17βHSD-1* promoter methylation in the breast cancer tissue specimens in women who had positive estrogen, progesterone receptors, and negative Her2 (Table 5).

### 3.3. The Concordant* BRCA1* and* 17βHSD-1* Promoter Methylation in Cancer and Normal Tissue Specimens

In addition, the concordant promoter methylation of the two studied genes was also investigated (Tables 6 and 7).* BRCA1* promoter was methylated in both normal and cancer breast tissue specimens of 11 patients which was statistically significant (*p* < 0.001), while 20 cases showed combined unmethylation of* BRCA1* promoter (Table 6).

In the case of a* 17βHSD-1* promoter, 37 tissue specimens had methylated normal and cancer tissues, while none of the studied tissues showed combined unmethylation of this gene promoter (Table 7).

### 3.4. Combined* BRCA1* and* 17βHSD-1* Promoter Methylation in Both Study Groups

Among the studied samples, 45% of cancer tissues and 35% of normal tissues had combined* BRCA1* and* 17βHSD-1* promoter methylation (Table 8). However, there was no significant relation between combined* BRCA1* and* 17βHSD-1* promoter methylation and type of tissue. The odds ratio = 1.5; 95% confidence interval = 0.6–3.7 and *p* = 0.361.

## 4. Discussion

*BRCA1* promoter hypermethylation is implicated as one of the mechanisms of loss of gene expression [33]. It was identified in 9–44% of sporadic breast cancers [19–21]. The mitogenic effect of estradiol on breast epithelium is counteracted by the upregulation of* BRCA1* expression, which in turn exerts a negative feedback effect on estradiol action by direct interaction with estrogen receptor [30]. Hence, transcriptional inactivation of* BRCA1* due to methylation may fail to decrease estradiol activity resulting in increased breast tissue proliferation. Despite the ample studies on the role of* BRCA1* in BC and its epigenetic modification, nevertheless, its association with the estrogen level regulating gene* 17βHSD-1* has not been conducted. In this study, we investigated the methylation status of* BRCA1* and* 17βHSD-1* in sporadic breast cancer Egyptian patients and correlated the findings to those in normal breast tissues.

*BRCA1* methylation percentage in our study is near to the upper end of previously reported frequencies for this alteration in sporadic breast cancer [19–21]. Hsu et al. data had hypermethylation of the* BRCA1* in 56% (78 of 139) of Taiwanese women with early-stage sporadic breast carcinomas, which is significantly higher than previously reported frequencies for this alteration in sporadic breast tumors [34]. The incidence of* BRCA1* methylation has previously been reported to be higher in breast tumors of infiltrating ductal type suggesting that it might play a role in breast carcinogenesis [19]. In other studies,* BRCA1* existed in even lower percentages, 9–32%, in sporadic breast cancer [33]. This variation may be due to several factors: first factor is the methylation assay or the analysis method used [35], bisulfite method [15, 19, 36, 37], and genomic sequencing [22, 38, 39]; secondly, MSP detects differential methylation status by amplification of bisulfite-treated DNA with primers specific for methylated versus unmethylated DNA [40]. CpG sites residing within the primer sets were used as a proxy for the methylation status of the region of interest. Although most published studies mentioned above [15, 19, 36, 37] used MSP, the primer sequences and target regions varied from study to study. Finally, contaminating unmethylated normal tissue may occur during tissue dissection that might attenuate the methylation levels of the tumor tissue.

A small population of apparently normal breast epithelial cells could harbor* BRCA1* promoter methylation in patients' samples with* BRCA1*-methylated tumors [41, 42], but not in those with* BRCA1*-unmethylated tumors.* BRCA1* promoter was methylated in both normal and cancer breast tissue specimens of 11 patients which was statistically significant (*p* < 0.001), while 20 cases showed combined unmethylation of* BRCA1* promoter. In the case of* 17βHSD-1* promoter, 37 tissue specimens had methylated normal and cancer tissues, while none of the studied tissues showed combined unmethylation of this gene promoter.

*BRCA1* methylation was detected in 76.5% of cancer tissues with positive ER and 64.7% with positive PR; on the other hand, methylation was significantly (*p* = 0.007) higher among tissues with negative Her2 (68.4%) than those with positive Her2, while* 17βHSD-1* promoter methylation was found in cancer tissue specimens in women who had positive ER (66.7%), positive PR (64.1%), and negative Her2 (46.2%).

Although we could not have complete data for the ER, PR, and Her2 receptor staining status for the entire studied sample size, our data showed that “luminal A” molecular subtype, defined as being positive for both ER and PR but negative for Her2 receptor, had the highest level of promoter methylation. In particular,* 17βHSD-1* showed the highest association with “luminal A” subtype. Similarly, methylation of* BRCA1* promoter correlated significantly with “luminal A” subtype and was evenly distributed among other molecular subtypes. Our finding that* 17βHSD-1* is associated with the luminal subtypes A and B is surprising since it has been shown that this enzyme activity correlates with the positivity of both ER and PR [28]. Methylation of* 17βHSD-1* in both normal and cancer tissue specimens directs the attentions towards saving those patients from the long term use of adjuvant antiestrogen therapies. Although our results indicated that* BRCA1* promoter methylation did not correlate significantly with triple-negative breast cancer (*p* = 0.174), Bal et al. showed that* BRCA1* promoter methylation correlated with decreased expression of ER and basal-like phenotype [33]. More than half of the patients with the* BRCA1* mutation had triple-negative breast cancer, and they also shared common clinical and pathological features [43]. However, a significant portion of triple-negative breast cancer patients does not carry* BRCA1* mutations. In studies of US and British women, triple-negative/basal-like tumors appeared to be more common among black women (especially before menopause) compared to white women [44, 45].

Theoretically, unmethylated/hypomethylated* 17βHSD-1* should increase the levels of active estradiol and the risk of BC. Contrary to this, we observed that methylation of* 17βHSD-1* was seen in 97.5% of tumor tissue compared to 95% of neighboring normal tissue. Some genes are known to alter their methylation status with age and these are usually tissue-specific [33]. Our results showed that the mean age of sporadic breast cancer patients with* 17βHSD-1* promoters methylation group (56.0 ± 9.3 years) was slightly higher than unmethylated group (49.0 years), although the relation between age and* 17βHSD-1* methylation was statistically insignificant. The present study, therefore, indicates that the methylation status of* 17βHSD-1* may be age-specific; alteration of this methylation pattern of the* 17βHSD-1* gene in breast tissue may play an important role in BC progression.

Our preliminary results presented here only demonstrate the association of* BRCA1* promoter methylation between tumors and normal breast tissues. Direct evidence shows that progression from* BRCA1*-methylated normal breast epithelial cells to* BRCA1*-methylated breast cancer needs to be investigated in future studies. The high methylation of* 17βHSD-1* promoter in both normal and cancer breast tissues rules out the biological significance of this epigenetic modification in distinguishing normal and cancer tissues. Given the fact that breast tumors express high heterogeneity, one limitation in explaining this finding is the small sample size of our studied population. Suzuki et al. showed that 17*β*HSD-1 negative breast tissues are less differentiated; hence, they escape normal regulation of proliferation; thus, it is possible that the reduced expression of this enzyme via hypermethylation in normal tissue reflects an increased carcinogenic potential in normal breast tissues harboring a nearby cancer tumor [28]. In addition, based on the fact that the activity of both* 17βHSD-2* and* 17βHSD-1* controls the in situ level of breast estrogen, it is possible that* 17βHSD-2* activity is dominating the control of estrogen level in the* 17βHSD-1* hypermethylated tumors and it would be beneficial to evaluate the methylation status of this gene especially in breast cancer hypermethylated* 17βHSD-1* tissues [46]. Furthermore, we found that methylation of* 17βHSD-1* in both normal and cancer tissue specimens may direct the attentions towards saving those patients from the long term use of adjuvant antiestrogen therapies, for example, tamoxifen, especially in ER^+^. Increased understanding of the genetic/epigenetic abnormality in the pathogenesis of breast cancer is crucial and may provide a basis for detection and treatment. This study highlights the frequent promoter methylation of* BRCA1* and its prognostic significance, irrespective of* BRCA1* gene mutation in Egyptian patients with early-stage breast cancer.

In conclusion, we showed that a significant proportion of patients with* BRCA1*-methylated tumors harbored* BRCA1* promoter methylation in normal breast tissues and that* 17βHSD-1* methylation was observed in the normal tissues of the* 17βHSD-1* promoter methylation status of the tumors. This suggests a possibility that a small proportion of the epithelial cells with* BRCA1* promoter methylation can be precursor cells from which* BRCA1*-methylated breast tumors originate. Although our preliminary results presented here need to be validated by future studies, they may provide further insight into the different roles of promoter methylation of these genes in breast carcinogenesis.

## Figures and Tables

**Table 1:** Primers used for PCR amplification after HpaII digestion, along with the target band size and annealing temperatures.

Gene	Primers sequence (5′–3′)	Annealing temperature	Amplicon size	Reference
*BRCA1*	F: TTGGGAGGGGGCTCGGGCAT	58°C	500 bp	[32]
	R: CAGAGCTGGCAGCGGACGGT			

*17βHSD-1 *	F: AGACCATCCTCACCAACAGG	55°C	238 bp	[32]
	R: CCTGGCCCTGTCATTTTTAG			

**Table 2:** Association of *BRCA1* promoter methylation with clinicopathological parameters.

	Overall (*N* = 40)	%	*BRCA1* methylation in normal tissues					*BRCA1* methylation in cancer tissues				
			Methylated (14)		Unmethylated (26)		*p* value	Methylated (17)		Unmethylated (23)		*p* value
			Freq.	(%)	Freq.	(%)		Freq.	(%)	Freq.	(%)	
*Age*:												
<50	10	25%	6	(42.9)	4	(15.4)	0.123	5	(29.4)	5	(21.7)	0.717
≥50	30	75%	8	(57.1)	22	(84.6)		12	(70.6)	18	(78.3)	
*Menopausal status*:												
Premenopause	7	17.5%	5	(35.7)	2	(7.7)		5	(29.4)	2	(8.7)	0.113
Postmenopause	33	82.5%	9	(64.3)	24	(92.3)		12	(70.6)	21	(91.3)	
*Age at menopause*:												
≤50	28	70.0%	8	(57.1)	20	(76.9)	0.079	10	(58.8)	18	(78.3)	0.317
>50	5	12.5%	1	(7.1)	4	(15.4)		2	(11.8)	3	(13.0)	
Premenopausal	7	17.5%	5	(35.7)	2	(7.7)		5	(29.4)	2	(8.7)	
Mean ± SD	49.2 ± 3.7		50.1 ± 2.8		48.8 ± 3.9		0.318	50.7 ± 3.1		48.3 ± 3.4		0.048^*∗*^
*Tumor size*:												
T1	4	10.0%	2	(14.3)	2	(7.7)	0.478	1	(5.9)	3	(13.0)	0.800
T2	28	70.0%	11	(78.6)	17	(65.4)		13	(76.5)	15	(65.2)	
T3	4	10.0%	1	(7.1)	3	(11.5)		2	(11.8)	2	(8.7)	
T4	4	10.0%	0	(0)	4	(15.4)		1	(5.9)	3	(13.0)	
*Lymph node status * **:**												
N0	11	27.5%	5	(35.7)	6	(23.1)	0.478	5	(29.4)	6	(26.1)	0.544
N1	8	20.0%	4	(28.6)	4	(15.4)		5	(29.4)	3	(13.0)	
N2	13	32.5%	3	(21.4)	10	(38.5)		4	(23.5)	9	(39.1)	
N3	8	20.0%	2	(14.3)	6	(23.1)		3	(17.6)	5	(21.7)	
*Clinical stage*:												
I	2	5.0%	2	(14.3)	0	(0.0)	0.124	1	(5.9)	1	(4.3)	0.940
IIA	10	25.0%	4	(28.6)	6	(23.1)		4	(23.5)	6	(26.1)	
IIB	13	32.5%	6	(42.9)	7	(26.9)		7	(41.2)	6	(26.1)	
IIIA	4	10.0%	0	(0.0)	4	(15.4)		1	(5.9)	3	(13.0)	
IIIB	3	7.5%	0	(0.0)	3	(11.5)		1	(5.9)	2	(8.7)	
IIIC	8	20.0%	2	(14.3)	6	(23.1)		3	(17.6)	5	(21.7)	
*Histological grade*:												
G1	3	7.5%	1	(7.1)	2	(7.1)	0.319	2	(11.8)	1	(4.3)	0.281
G2	30	75.0%	11	(78.6)	19	(73.1)		14	(82.4)	16	(69.6)	
G3	3	7.5%	2	(14.3)	1	(3.8)		1	(5.9)	2	(8.7)	
No grade	4	10.0%	0	(0.0)	4	(15.4)		0	(0.0)	4	(17.4)	

^*∗*^Chi-square exact test is statistically significant at 95% confidence level.

**Table 3:** Association of *17βHSD-1* promoter methylation with clinicopathological parameters.

	Overall (*N* = 40)	%	*17βHSD-1* methylation in normal tissues					*17βHSD-1* methylation in cancer tissues				
			Methylated (38)		Unmethylated (2)		*p* value	Methylated (39)		Unmethylated (1)		*p* value
			Freq.	(%)	Freq.	(%)		Freq.	(%)	Freq.	(%)	
*Age*:												
<50	10	25%	5	(29.4)	5	(21.7)	0.717	9	(23.1)	1	(100.0)	0.250
≥50	30	75%	12	(70.6)	18	(78.3)		30	(76.9)	0	(0.0)	
*Menopausal status*:												
Premenopause	7	17.5%	7	(18.4)	0	(0.0)	1.000	7	(17.9)	0	(0.0)	1.000
Postmenopause	33	82.5%	31	(81.6)	2	(100.0)		32	(82.1)	1	(100.0)	
*Age at menopause*:												
≤50	28	70.0%	26	(68.4)	2	(100.0)	0.637	27	(69.2)	1	(100.0)	1.000
>50	5	12.5%	5	(13.2)	0	(0.0)		5	(12.8)	0	(0.0)	
Premenopausal	7	17.5%	7	(18.4)	0	(0.0)		7	(17.9)	0	(0.0)	
Mean ± SD	49.2 ± 3.7		49.5 ± 3.4		44 ± 5.7		0.397	49.2 ± 3.7		45.0		0.242
*Tumor size*:												
T1	4	10.0%	4	(10.5)	0	(0.0)	0.271	3	(7.7)	1	(100.0)	0.300
T2	28	70.0%	27	(71.1)	1	(50.0)		28	(71.8)	0	(0.0)	
T3	4	10.0%	3	(7.9)	1	(50.0)		4	(10.3)	0	(0.0)	
T4	4	10.0%	4	(10.5)	0	(0.0)		4	(10.3)	0	(0.0)	
*Lymph node status*:												
N0	11	27.5%	11	(28.9)	0	(0.0)	0.368	11	(28.2)	0	(0.0)	0.400
N1	8	20.0%	7	(18.4)	1	(50.0)		7	(17.9)	1	(100.0)	
N2	13	32.5%	13	(34.2)	0	(0.0)		13	(33.3)	0	(0.0)	
N3	8	20.0%	7	(18.4)	1	(50.0)		8	(20.5)	0	(0.0)	
*Clinical stage*:												
I	2	5.0%	2	(5.3)	0	(0.0)	0.327	2	(5.1)	0	(0.0)	0.675
IIA	10	25.0%	10	(26.3)	0	(0.0)		9	(23.1)	1	(100.0)	
IIB	13	32.5%	13	(34.2)	0	(0.0)		13	(33.3)	0	(0.0)	
IIIA	4	10.0%	3	(7.9)	1	(50.0)		4	(10.3)	0	(0.0)	
IIIB	3	7.5%	3	(7.9)	0	(0.0)		3	(7.7)	0	(0.0)	
IIIC	8	20.0%	7	(18.4)	1	(50.0)		8	(20.5)	0	(0.0)	
*Histological grade*:												
G1	3	7.5%	3	(7.9)	0	(0.0)	0.873	3	(7.7)	0	(0.0)	1.000
G2	30	75.0%	28	(73.7)	2	(100.0)		29	(74.4)	1	(100.0)	
G3	3	7.5%	3	(7.9)	0	(0.0)		3	(7.7)	0	(0.0)	
No grade	4	10.0%	4	(10.5)	0	(0.0)		4	(10.3)	0	(0.0)	

Chi-square exact test is statistically significant at 95% confidence level.

**Table 4:** Association of *BRCA1* promoter methylation with mitosis, immunohistochemistry, and molecular subtype of breast cancer.

	Overall (*N* = 40)	%	*BRCA1* methylation in normal tissues					*BRCA1* methylation in cancer tissues				
			Methylated (14)		Unmethylated (26)		*p* value	Methylated (17)		Unmethylated (23)		*p* value
			Freq.	(%)	Freq.	(%)		Freq.	(%)	Freq.	(%)	
*Mitosis*:												
0	4	10.0%	0	(0)	4	(15.4)	0.292	0	(0.0)	4	(17.4)	0.042^*∗*^
1	5	12.5%	3	(21.4)	2	(7.7)		4	(23.5)	1	(4.3)	
2	29	72.5%	10	(71.4)	19	(73.1)		13	(76.5)	16	(69.6)	
3	2	5.0%	1	(7.1)	1	(3.8)		0	(0.0)	2	(8.7)	
*Estrogen receptor*:												
Positive	27	67.5%	10	(71.4)	17	(65.4)	0.912	13	(76.5)	14	(60.9)	0.568
Negative	7	17.5%	2	(14.3)	5	(19.2)		2	(11.8)	5	(21.7)	
No available data	6	15.0%	2	(14.3)	4	(15.4)		2	(11.8)	4	(17.4)	
*Progesterone receptor*:												
Positive	25	62.5%	9	(64.3)	16	(61.5)	0.985	11	(64.7)	14	(60.9)	0.907
Negative	9	22.5%	3	(21.4)	6	(23.1)		4	(23.5)	5	(21.7)	
No available data	6	15.0%	2	(14.3)	4	(15.4)		2	(11.8)	4	(17.4)	
*Her2*:												
Positive	10	25.0%	2	(14.3)	8	(30.8)	0.279	2	(11.8)	8	(34.8)	0.007^*∗*^
Negative	19	47.5%	9	(64.3)	10	(38.5)		13	(76.4)	6	(26.1)	
No available data	11	27.5%	3	(21.4)	8	(30.8)		2	(11.8)	9	(39.1)	
*Molecular subtype*:												
Luminal A	13	32.5%	7	(50.0)	6	(23.1)	0.242	9	(52.9)	4	(17.4)	0.049^*∗*^
Luminal B	9	22.5%	2	(14.3)	7	(26.9)		2	(11.8)	7	(30.4)	
Basal-like	2	5.0%	1	(7.1)	1	(3.8)		2	(11.8)	0	(0.0)	
Her2	1	2.5%	1	(7.1)	0	(0.0)		0	(0.0)	1	(4.3)	
Others	8	20.0%	1	(7.1)	7	(26.9)		2	(11.8)	6	(26.1)	
No available data	7	17.5%	2	(14.3)	5	(19.2)		2	(11.8)	5	(21.7)	

^*∗*^Chi-square exact test is statistically significant at 95% confidence level.

**Table 5:** Association of *17βHSD-1* promoter methylation with mitosis, immunohistochemistry, and molecular subtype of breast cancer.

	Overall (*N* = 40)	%	*17βHSD-1* methylation in normal tissues					*17βHSD-1* methylation in cancer tissues				
			Methylated (38)		Unmethylated (2)		*p* value	Methylated (39)		Unmethylated (1)		*p* value
			Freq.	(%)	Freq.	(%)		Freq.	(%)	Freq.	(%)	
*Mitosis*:												
0	4	10.0%	4	(10.5)	0	(0.0)	0.850	4	(10.3)	0	(0.0)	1.000
1	5	12.5%	5	(13.2)	0	(0.0)		5	(12.8)	0	(0.0)	
2	29	72.5%	27	(71.1)	2	(100.0)		28	(71.8)	1	(100.0)	
3	2	5.0%	2	(5.3)	0	(0.0)		2	(5.1)	0	(0.0)	
*Estrogen receptor*:												
Positive	27	67.5%	25	(65.8)	2	(100.0)	0.602	26	(66.7)	1	(100.0)	1.000
Negative	7	17.5%	7	(18.4)	0	(0.0)		7	(17.9)	0	(0.0)	
No available data	6	15.0%	6	(15.8)	0	(0.0)		6	(15.4)	0	(0.0)	
*Progesterone receptor*:												
Positive	25	62.5%	23	(60.5)	2	(100.0)	0.532	25	(64.1)	0	(0.0)	0.375
Negative	9	22.5%	9	(23.7)	0	(0.0)		8	(20.5)	1	(100.0)	
No available data	6	15.0%	6	(15.8)	0	(0.0)		6	(15.4)	0	(0.0)	
*Her2*:												
Positive	10	25.0%	10	(26.3)	0	(0.0)	0.312	10	(25.6)	0	(0.0)	1.000
Negative	19	47.5%	17	(44.7)	2	(100.0)		18	(46.2)	1	(100.0)	
No available data	11	27.5%	11	(29.0)	0	(0.0)		11	(28.2)	0	(0.0)	
*Molecular subtype*:												
Luminal A	13	32.5%	11	(28.9)	2	(100.0)	0.120	13	(32.5)	0	(0.0)	0.174
Luminal B	9	22.5%	9	(23.7)	0	(0.0)		9	(22.5)	0	(0.0)	
Basal-like	2	5.0%	2	(5.3)	0	(0.0)		2	(5.1)	0	(0.0)	
Her2	1	2.5%	1	(2.6)	0	(0.0)		1	(2.6)	0	(0.0)	
Others	8	20.0%	8	(21.1)	0	(0.0)		7	(17.9)	1	(100.0)	
No available data	7	17.5%	7	(18.4)	0	(0.0)		7	(17.9)	0	(0.0)	

Chi-square exact test is statistically significant at 95% confidence level.

**Table 6:** Relation between methylation status of *BRCA1* promoter in cancer tissue specimens and normal tissue specimens in 40 sporadic breast cancer patients.

		*BRCA1* cancer tissues							
		Methylated		Unmethylated		Total		*p* value	OR (95% CI)
		Freq.	(%)	Freq.	(%)	Freq.	%		
*BRCA1* normal tissues	Methylated	11	(78.6)	3	(21.4)	14	100.0	0.001^*∗*^	12.2 (2.5–58.7)
	Unmethylated	6	(23.1)	20	(76.9)	26	100.0		
	Total	17	(42.5)	23	(57.5)	40	100.0		

^*∗*^Chi-square test is statistically significant at 95% confidence level.

**Table 7:** Relation between methylation status of *17βHSD-1* promoter in cancer tissue specimens and normal tissue specimens in 40 sporadic breast cancer patients.

		*17βHSD-1* cancer tissues						
		Methylated		Unmethylated		Total		*p* value
		Freq.	(%)	Freq.	(%)	Freq.	%	
*17βHSD-1* normal tissues	Methylated	37	(97.4)	1	(2.6)	38	100.0	1.000
	Unmethylated	2	(100.0)	0	(0.0)	2	100.0	
	Total	39	(97.5)	1	(2.5)	40	100.0	

Chi-square test is statistically significant at 95% confidence level.

**Table 8:** Relation between combined *BRCA1* and *17βHSD-1* promoter methylation and the type of breast tissues of 40 sporadic breast cancer patients.

Combined *BRCA1* and *17βHSD-1* promoter methylation								
	Overall (*N* = 40)		Methylated		Unmethylated		*p* value	OR (95% CI)
	Freq.	%	Freq.	(%)	Freq.	(%)		
*Type of tissue*								
Cancer tissues	40	100.0%	18	(45.0)	22	(55.0)	0.361	1.5 (0.6–3.7)
Normal tissues	40	100.0%	14	(35.0)	26	(65.0)		
Total	80	100.0%	32	(40.0)	48	(60.0)		

Chi-square test is statistically significant at 95% confidence level.
